# Veterinary College Notes

**Published:** 1890-03

**Authors:** 


### VETERINARY COLLEGE NOTES.

*Ontario Veterinary College.*—In considering the educational forces operating in connection with any college, the various societies for professional work must not be overlooked. Few things in a student's life assist him better to make a success of his work than to take an active part in such societies.

No doubt every veterinary college has its own society, or societies, for the reading and discussion of papers by its own students, but it is of that in connection with the Ontario Veterinary College we wish now to speak.

As to the date of its organization, we cannot state at this time, but in looking over the Secretary's minute book we find the first meeting there entered was held in October, 1883. The roll-call of this meeting shows 104 members present. G. W. Butler, V.S., of Circleville, Ohio; J. Wende, V.S., of Buffalo, N. Y.; A. Harthill, V.S., of Louisville, Ky., were among the officers. All of these gentlemen, although they have distinguished themselves as practitioners since that time, were then students. In fact, every officer of this society (save the President, Professor Smith), is elected from the students themselves.

The average attendance during that session was about 120, which gives some idea of the interest attaching to those meetings, most of the students being present at each meeting, which were held twice a week. This was in 1883, and every session since has witnessed greater interest and increased attendance.

During last session, 1888–89, the meetings were held on the evenings of Tuesday and Friday. Owing to the numbers present, roll-call was omitted, it being found that the time occupied by calling over 400 names could be more profitably occupied. More than 200 papers were read and discussed last year, many of the papers being of great merit, some being considered worthy of publication in the *Veterinary Journal* of London. We hope to present some notices of these meetings this session, with some of the papers read.

